# A protocol for a systematic review of the use of process evaluations in knowledge translation research

**DOI:** 10.1186/2046-4053-3-149

**Published:** 2014-12-23

**Authors:** Shannon D Scott, Thomas Rotter, Lisa Hartling, Thane Chambers, Katherine H Bannar-Martin

**Affiliations:** Faculty of Nursing, Level 3, Edmonton Clinic Health Academy, University of Alberta, 11405 87 Avenue, Edmonton, Alberta T6G 1C9 Canada; ECHO (Transferring Evidence in Child Health to enhance Outcomes), Edmonton, Canada; Health Quality Improvement Sciences, College of Pharmacy and Nutrition, University of Saskatchewan, 226 Thorvaldson Building, Saskatoon, Saskatchewan S7N 5C9 Canada; Alberta Research Centre for Health Evidence Department of Pediatrics, University of Alberta, 9424 Aberhart Centre, Edmonton, AB T6G 2J3 Canada; Faculty of Nursing, Public Services Librarian, JW Scott Health Sciences Library, University of Alberta, Edmonton, Alberta Canada; Faculty of Nursing, University of Alberta, Edmonton, Alberta Canada

**Keywords:** Process evaluation, Knowledge translation, Research use, Health interventions, Systematic review

## Abstract

**Background:**

Experimental designs for evaluating knowledge translation (KT) interventions for professional behavior change can provide strong estimates of intervention effectiveness but offer limited insight how the intervention worked or not. Furthermore, trials provide little insight into the ways through which interventions lead to behavior change and how they are moderated by different facilitators and barriers. As a result, the ability to generalize the findings from one study to a different context, organization, or clinical problem is severely compromised. Consequently, researchers have started to explore the causal mechanisms in complementary studies (process evaluations) alongside experimental designs for evaluating KT interventions. This study focuses on improving process evaluations by synthesizing current evidence on process evaluations conducted alongside experimental designs for evaluating KT interventions.

**Methods/Design:**

A medical research librarian will develop and implement search strategies designed to identify evidence that is relevant to process evaluations in health research. Studies will not be excluded based on design. Included studies must contain a process evaluation component aimed at understanding or evaluating a KT intervention targeting professional behavior change. Two reviewers will perform study selection, quality assessment, and data extraction using standard forms. Disagreements will be resolved through discussion or third party adjudication. Data to be collected include study design, details about data collection approaches and types, theoretical influences, approaches to evaluate intervention dose delivered, intervention dose received, intervention fidelity, intervention reach, data analysis, and study outcomes. This study is not registered with PROSPERO.

**Discussion:**

There is widespread acceptance that the generalizability of quantitative trials of KT interventions would be significantly enhanced to other contexts, health professional groups, and clinical conditions through complementary process evaluations alongside trials. This systematic review will serve as a ‘state of the science’ on methodological approaches to process evaluations and will allow us to: 1) take stock of current research approaches and 2) develop concrete recommendations for knowledge users (e.g., quality consultants and health services researchers) designing future KT process evaluations.

**Electronic supplementary material:**

The online version of this article (doi:10.1186/2046-4053-3-149) contains supplementary material, which is available to authorized users.

## Background

It is well established that implementing research into practice is a complex undertaking [1] that requires taking into account multiple levels such as the patient, health care provider, multidisciplinary team, health care institution, and local and national health care systems. Furthermore, given all of these complexities, it is increasingly clear that shifting towards implementation of proven treatments (rather than a continued, almost exclusive, focus on developing new treatments) is crucially important. As a result, the improvement of health services and a stronger, more robust health care system critically rests on the development and evaluation of interventions to implement evidence-informed knowledge. It is increasingly recognized that improving the content and availability of research is not enough to facilitate implementation [2]; rather, explicit and active interventions (known as knowledge translation interventions or strategies) are essential to facilitate knowledge translation.

Rigorous experimental designs for evaluating knowledge translation (KT) interventions to implement research can provide strong estimates of intervention effectiveness but offer limited insight into how the intervention worked or not, as well as how the intervention could be improved in the future [1]. Furthermore, experimental designs provide little insight into the ways through which interventions lead to implementation and how they are moderated by different facilitators and barriers. As a result, the ability to generalize the findings from one study to a different context, organization, or clinical problem is severely compromised. To remedy this, researchers have started to explore the causal mechanisms in complementary studies (e.g., process evaluations) alongside experimental designs for evaluating KT interventions. Until now, there have been no standards or guidance, specific to knowledge translation interventions, to guide the explicit design (e.g., research design, data collection types, and time points for examples) of these complementary yet vitally important process evaluations. This lack of standardization has hindered the generalizability of this research while simultaneously making cross-study comparisons problematic and designing process evaluations in KT research even more challenging. The aim of this project is to synthesize the evidence on extant process evaluations conducted alongside experimental designs for evaluating KT interventions to make recommendations for multiple end-user groups. This knowledge is critically important as health care providers, health quality consultants, decision and policy makers, NGOs, governmental departments, partnerships, and health services researchers have a responsibility to evaluate the effectiveness of their KT efforts to ensure that scarce health care resources are effectively utilized as well as ensure enhanced generalizability of their knowledge to benefit others around the globe.

Experimental designs such as randomized trials, cluster randomized trials, and stepped wedge designs are widely used, yet sometimes contentiously debated [3–5], designs in the knowledge translation field for evaluating the effectiveness of various interventions to implement research. These designs can provide a measure of the effectiveness of the intervention, specifically an outcome. The challenge is that the knowledge translation interventions and the health settings where the research occurs are complex, context-laden, and difficult, if not impossible to standardize. Knowledge translation interventions can target different audiences (e.g., health care providers, decision makers) and intervention scope including financial interventions, educational interventions, organizational interventions, and regulatory interventions, for example. Commonly used KT interventions include strategies such as distribution of educational materials, harnessing the influence of local opinion leaders, audit and feedback, and reminders, either used as single interventions or combined into multi-faceted interventions with multiple components.

It is particularly challenging to evaluate the effectiveness of knowledge translation interventions [3–5] because they contain several interacting components, such as the degree of flexibility or tailoring of the intervention, the number of interacting components within the interventions, and the number and difficulty of behaviors required by those delivering or receiving the intervention [3]. As a result, KT interventions pose methodological challenges and require augmentations to the standard experimental designs [6]. In 2000, the Medical Research Council in the United Kingdom released the evaluation framework for designing and evaluating complex interventions [4]; the framework was later revised in 2008 [7]. This document draws attention to the daunting task of both designing and evaluating complex interventions, such as KT interventions, through description of four phases that reflect the evolution of an intervention from theory through to long-term intervention implementation. The framework, however, does not recommend specific evaluation research designs and furthermore does not identify specific data collection types, time points, and standardized approaches for evaluating KT intervention dose, reach, and fidelity. This level of specificity is urgently required to compare across KT intervention evaluations and to understand how change is being effected and which factors mediate implementation.

The effectiveness of KT interventions in real-world clinical settings is dependent on many factors (e.g., context and mechanisms) other than the intervention itself [4]. Thus, other approaches to evaluation are required. Process evaluations, as stand-alone studies or nested alongside experimental designs, explore the ways that the KT intervention is implemented [8]. In the literature, ‘process’ and ‘qualitative’ are often used interchangeably [6], yet data for process evaluations can be both qualitative and quantitative. Process evaluations can be used to assess the fidelity and quality of implementation [9, 10] and identify the causal mechanisms [11, 12] and contextual factors associated with variation in outcomes across sites [6, 13]. Furthermore, process evaluations can assist in interpreting the outcome results [8], the barriers and facilitators to implementation [14, 15], and sustainability [16] as well as examining the participants’ views [17] and understandings of components of the intervention [18, 19]. If knowledge translation interventions had consistent effects across different contexts, conditions, and provider groups that could be generalized, then this would not be an issue. However, it is widely accepted that the effectiveness of KT interventions appears to vary across different contexts (settings), professional groups, and clinical conditions presumably because the causal mechanisms of the interventions (e.g., attributes of the interventions, dose delivered, dose received, reach, and fidelity) are modified in the presence of different barriers and facilitators in each setting. These causal mechanisms and effect modifiers shaping the implementation process have not been systematically studied or understood. Furthermore, generalizing findings from exclusive experimental designs into routine health care settings is highly problematic given the limited understanding of how the causal mechanisms and effect modifiers in the implementation process work. As Eccles and colleagues [20] suggest, ‘it is an expensive version of trial and error, with no *a priori* reason to expect success (an intervention with a positive effect) or to have confidence of being able to replicate success if it is achieved’. Thus, process evaluations alongside experimental evaluations of KT interventions are critical to facilitating future implementation success through understanding how the effect has been achieved and sustained. This understanding is of the utmost significance given the limited health care resources and the reality that health care advancements are occurring exponentially, and there is increasing pressure to ensure that these advancements are implemented to improve health care outcomes and health delivery.

Variability in intervention effectiveness across contexts, mechanisms, and clinical problems makes it critical that both process (qualitative and mixed method) and outcome (quantitative experimental) evaluations are provided. The results of an experimental design (outcome evaluation) can only indicate if the intervention was effective or not. However, a lack of intervention effect may in fact be implementation failure rather than genuine ineffectiveness of the intervention. Process evaluations are vital in identifying the success or failure of implementation, which is critical in understanding intervention effectiveness.

Until now, there have been no definitive standards to guide the design and development of process evaluations conducted alongside experimental evaluations of KT interventions. Just recently, Grant and colleagues [21] proposed a framework for process evaluations for design-specific cluster-randomized trials of complex interventions; unfortunately, their recommendations were not based upon a comprehensive, systematic review of all approaches employed by others who have previously conducted these studies. As a result, this framework falls short of providing a state of-the science of these important investigations. In 2009, Lewin and colleagues conducted the only evaluation of qualitative approaches alongside randomized trials of complex health care interventions [22]. Through this work, they discovered that 30% of the randomized controlled trials had associated qualitative investigations. Of these studies, the qualitative work was largely completed before the trial with smaller numbers of studies completing qualitative work during the trial and following it. Lewin and colleagues also discovered that the qualitative work was completed for a range of rationales including explaining variation in effectiveness, exploring responses to the interventions, and understanding the change and implementation processes. Although this work is noteworthy, it is considerably limited by the methods used and its scope. The authors did not attempt to systematically review all of the process evaluations completed; rather, they selected a systematic sample of 100 trials published 2001–2003 by the Cochrane Effective Practice and Organisation of Care (EPOC) Review Group and investigated if these trials had a qualitative component. Furthermore, Lewin focused on qualitative components; thus mixed method process evaluations may not have consistently been included in his review, and this review was not specific to KT interventions but rather complex health care interventions in the EPOC database. Given the incomplete approach and the date restrictions of the trials included, it is critically important to systematically review all of the process evaluations of KT interventions. It is accepted that increasing numbers of experimental evaluations of interventions now include process evaluations; furthermore, there has been an exponential increase in KT research during the past decade thus further necessitating this review.

The findings from our proposed knowledge synthesis will respond to a significant gap in the literature and will provide critical information to guide the decision-making of 1) health care decision and policy makers who are charged with implementing knowledge into clinical practice in Canada and globally, 2) health care quality consultants who are charged with implementing evidence-informed clinical practice changes and evaluating their effectiveness, 3) non-governmental organizations (not-for-profit) and government departments implementing evidence-informed practices, and 4) researchers conducting process evaluations alongside KT interventions.

## Methods/Design

### Objectives and key questions

The objectives for this systematic review are to (1) systematically locate, assess, and report on knowledge translation studies in health that have a process evaluation component or are a stand-alone process evaluation of a KT intervention study; (2) describe the interface between the process evaluation findings (process) and the experimental findings (outcome/effectiveness) from the KT intervention (if any); and (3) offer guidance for researchers in terms of the development and design of process evaluations of KT interventions. These objectives will be accomplished by (1) identifying and describing the methodological design of the process evaluations; (2) identifying the data collection types, time points, and data analysis processes; (3) identifying if the process evaluations were informed by theory; and (4) identifying approaches used to evaluate the KT intervention dose delivered, intervention dose received, KT intervention fidelity, and intervention reach. In accordance with this review’s objectives, the key questions that will guide this systematic review are as follows: (1) what is the ‘state-of-the-science’ of process evaluations conducted alongside trials in knowledge translation (as either stand-alone studies or as a component of a KT trial); and (2) what is the effectiveness of various process evaluation designs used in knowledge translation?

### Methods

This systematic review will follow a comprehensive methodology using rigorous guidelines to synthesize diverse forms of research evidence [23]. Although some controversy exists regarding the legitimacy of synthesizing various research methodologies (e.g., quantitative and qualitative), an exclusive reliance on research studies employing controlled clinical trials (CCTs), controlled before and after (CBA), randomized control trials (RCTs) studies, and interrupted time series (ITS) designs may not reflect the intricacies of the different types of ‘evidence’ utilized to guide decision making [24]. There is a growing recognition that the complexities inherent in evidence cannot be captured exclusively through a single methodology [20, 24, 25]. Therefore, to respond to the needs of ‘decision makers’ and to acknowledge the diverse landscape of the process evaluation literature, this project will favor methodological inclusivity rather than exclusivity. Consequently, our review will combine conventional approaches to systematic reviews with methods for accommodating different study designs (e.g., qualitative studies and mixed method studies) present in selected studies. This study is not registered with PROSPERO.

### Literature search

A health research librarian (with information science training), in collaboration with the research team, will create, revise (as needed), and implement search strategies designed to identify relevant evidence (Additional file 1). The design of the search strategy will also be peer-reviewed by another health research librarian familiar with the complexities of searching for knowledge translation literature. Preliminary search strategies indicate a significant amount of literature available to synthesize. To ensure an exhaustive search is conducted, a comprehensive set of subject headings and keywords will be used in a variety of databases. Language (English) and date (1996–2013) restrictions will be employed. We will systematically search the following electronic databases that store resources with this research-related focus: Ovid MEDLINE and In-Process & Other Non-Indexed Citations, Ovid EMBASE, Ovid PsycINFO, EBSCOhost CINAHL, and ISI Web of Science. Reference lists of included studies will be reviewed for further relevant citations. The PubMed database will also be searched for in-process and non-indexed publications.

### Study inclusion criteria

Studies will not be excluded based upon research design. While controversial, the inclusion of study designs other than exclusively RCT and quasi-experimental is particularly important in an emerging field without standard indexing terms, such as process evaluations of KT interventions. By including these designs, the results will reflect the rich and emerging literature base on process evaluations as well as generate hypotheses that could be tested in studies with more rigorous designs. The inclusion criteria (Table 1) will be used for study selection.

**Table 1:** **Process evaluation systematic review inclusion criteria**

Study design	Research studies including all designs, e.g., experimental, quasi-experimental, and non-experimental designs (e.g., case study). Opinion pieces, commentaries, methodological papers, book chapters, books, dissertations, conference abstracts, protocols, and reviews will not be included.
Study criteria	The study *is* or *includes a process evaluation* of a *health* implementation study/project or a health research implementation/KT study that has a primary purpose of translating research into action/practice. The health (research) information disseminated must therefore be evidence-based.^a^
	A *registered*/*licensed health care professional* or *allied health care professional* (in medicine (physician, dentist), nursing, rehabilitation medicine (physiotherapy, occupational therapy, speech-language pathology), dietetics, or pharmacy must either *deliver or receive the intervention* (*sensu* Scott et al. [26]).
	A trainee health care professional (not yet licensed/registered) either delivering or receiving the intervention will be excluded if:
	a. The intervention is mandatory curricula for finishing their degree/gaining licensing
	b. The intervention has no licensed health care professional involved.
Outcome(s)	The process evaluation component is distinct from the primary outcomes of the KT/research implementation component if both the process evaluation and KT implementation are reported in the study. Where the paper is only reporting the process evaluation it will be considered a separate outcome.

^a^Health is defined according to the WHO [27] conceptualization of a state of complete physical and mental well-being and not merely the absence of disease or infirmity, including prevention components and mental health but not ‘social health’.

### Study selection

A two-step process will be used for study screening. Titles and abstracts (when available) will be independently screened by two reviewers against the inclusion criteria (Table 1). Each article will be classified as ‘include’, ‘exclude’, or ‘unclear’. The full text of articles classified as ‘include’ or ‘unclear’ will be obtained and independently reviewed against the pre-determined inclusion criteria (Table 1), using a standard form in Microsoft Access (Additional file 2). A third-party adjudicator will be resolve discrepancies between the two reviewers by dialog.

### Quality assessment

The process for assessing the methodological quality of included studies will be informed by recommended processes within the emerging field of mixed studies reviews (i.e., examines quantitative, qualitative, and mixed methods primary studies together) in health sciences [21]. Included studies will be independently assessed by two independent reviewers. Discrepancies will be addressed through discussion between the two reviewers and third-party adjudication where necessary. Inter-rater agreement will be calculated using the weighted kappa statistic [28]. The methodological quality of all included studies will be assessed using Mixed Methods Appraisal Tool [28] (Additional file 3). The tool’s validity and reliability have been verified [29–31], and currently, the MMAT offers the best and most comprehensive tool for assessing studies of multiple method types. The results from the tool lead to an overall methodological score calculated as a percentage. The criteria used to determine the quality score varies by design. This tool has been previously tested for reliability and meets accepted standards [32].

### Data extraction

Study data will be extracted using standardized Microsoft Access forms (Additional file 4) and entered into database tables. All data extraction forms will be pre-programmed to guide reviewers through the data extraction process and help control the data quality. Data will be independent extracted by two independent reviewers for completeness and compared for accuracy and then compiled for completeness. Data indicators to be extracted include study design and process, details about data collection approaches and types, theoretical influences, approaches to evaluate KT intervention dose delivered, intervention dose received, intervention fidelity, intervention reach, data analysis, and study outcomes. The data extraction form will be trialed on 10 studies to refine the form and ensure the form captures all of the intricacies of qualitative, quantitative, multi method, and mixed method designs.

### Data analysis/synthesis

Foremost, the data extracted will be grouped and analyzed by study design (e.g., mixed method, and qualitative). Next, data will be aggregated and analyzed according to the type of KT intervention (e.g., the same types of interventions may use similar methods for process evaluations). From this analysis, we will present a descriptive analysis of the included studies and look at the patterns in terms of the design and effectiveness of the process evaluation approaches.

Evidence tables will be created to summarize and describe the studies included in this review. Variables to be evaluated in the descriptive analysis include 1) country of primary author, 2) study design, 3) quality assessment of studies, and 4) type and details of the process evaluation. A qualitative review across the studies will allow us to not only examine what designs are successful but also evaluate what it is about different process evaluation approaches that may work and under what circumstances [33]. The value of our review is that data will not just be synthesized to get an overall assessment of whether particular approaches to process evaluations are effective but rather assess the intricacies and details of these approaches. The results of our review will richly add to the evidence base as it goes beyond the results of a ‘typical’ systematic review.

### Integrated knowledge translation plan

#### Decision-maker and stakeholder partnerships

We have developed a strong Knowledge User Advisory Panel (headed by a Primary Knowledge User, TR) to ensure that our synthesis outputs respond to the information needs of stakeholders and knowledge users. The six chosen knowledge users reflect the multiple and relevant end users and audiences for this project (e.g., policy and decision makers, health care professionals) and we will utilize the expertise of the Knowledge User Advisory Panel to provide strategic advice throughout the research process. The research team and Knowledge User Advisory Panel will at minimum formally meet once in person (at midway of the project to share interim results) and once via teleconference (at the end of the project to further discuss ongoing dissemination and exchanges with key end users). The Knowledge User Advisory Panel is comprised of health care professionals and decision makers with a vested interest in evaluating research implementation efforts. Thus, their involvement in the co-creation of this knowledge is significant and meaningful to their day-to-day professional role.

The Knowledge User Advisory Panel will advise the research team on the strategic development of suitable ‘end products’ for the systematic review and a plan for disseminating end products to the appropriate local, national, and international groups and associations. Meaningful engagement with users of research by means of our Knowledge User Advisory Panel and our ongoing consultations with stakeholders from a broad range of organizations will ensure that 1) our research questions and project aims are relevant and applicable to issues or concerns to them locally, 2) project funds are used appropriately and judiciously, and 3) the findings inform innovative strategies to make a quality change in clinical practice, education, and research endeavors in terms of using effective process evaluations to determine the factors influencing the implementation of KT interventions.

#### Outcomes: end-of-grant knowledge translation

We will customize the research results to targeted user groups, including health care professionals, health care consumers, decision makers, and researchers. We will disseminate the finding of our process evaluation systematic review in media that are congruent with our findings, as guided by our Knowledge User Advisory Panel.

We will present our study results at health care research seminars and conferences, provide specific fact sheets, and meet face-to-face or communicate by phone to discuss the findings from the project. We will highlight practical strategies that could maximize use of non-traditional approaches in their specific setting. We will also circulate a one-page executive summary and project technical report that addresses the objectives of this research.

## Discussion

Health care systems around the world are faced with the challenges of improving quality of care and reducing the risk of adverse events. It is well established that health care systems fail to use the best available research to optimally inform health care. Health care system inefficiency and a reduction of quantity and quality of life is the outcome. As a result, there is much interest in KT; however, strong science is urgently needed to underpin and guide the current interest, activity, and investigation in the KT field. There is wide spread acceptance that the generalizability of quantitative trials of KT interventions would be significantly enhanced to other contexts, health professional groups, and clinical conditions through complementary process evaluations alongside trials. This systematic review will serve as a ‘state of the science’ on methodological approaches to process evaluations and will allow us to 1) take stock of current research approaches and 2) develop concrete recommendations for health quality consultants, health care professionals, health policy and decision makers, health services researchers, non-governmental organizations, and governmental departments implementing evidence-informed change in order to design future KT process evaluations.

## Authors’ information

SDS holds a Canada Research Chair for Knowledge Translation in Child Health, and is an AHFMR Population Health Investigator. TR holds a Saskatchewan Research Chair in Health Quality Improvement Science at the University of Saskatchewan. LH holds a CIHR New Investigator Award.

## Electronic supplementary material

Additional file 1: **Peer-reviewed search strategy.** Initial search strategy developed by medical research librarian. (DOCX 36 KB)

Additional file 2: **Standardized Microsoft Access study screening form.** Standardized Microsoft Access study screening form to be used to select studies for inclusion in this systematic review. (DOCX 198 KB)

Additional file 3: **Mixed methods appraisal tool (MMAT).** Mixed methods quality assessment tool to be used to assess methodological quality of included research studies. (PDF 93 KB)

Additional file 4: **Standardized Microsoft Access data extraction form.** Standardized Microsoft Access data extraction form to be used to extract data from included studies. (DOCX 327 KB)

## Abbreviations

KT: knowledge translation
CBA: controlled before and after design
CCT: clinical controlled trial
ITS: interrupted time series design
RCT: randomized controlled trial.
