# Supplementary figures and images for: A protocol for a systematic review of the use of process evaluations in knowledge translation research
Source: Syst Rev. 2014 Dec 23;3:149. doi: 10.1186/2046-4053-3-149 (PMC4307977; doi:10.1186/2046-4053-3-149)

**Additional File 2:**


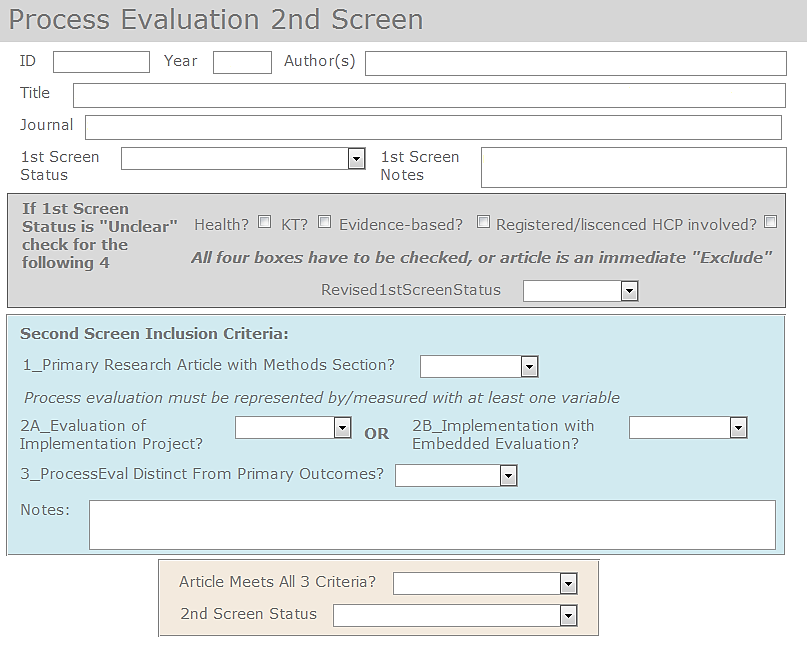

Supplement: Supplementary file 2 — Additional file 2: Standardized Microsoft Access study screening form. Standardized Microsoft Access study screening form to be used to select studies for inclusion in this systematic review. (DOCX 198 KB) [file 13643_2014_316_MOESM2_ESM.docx]

**Additional File 4**:


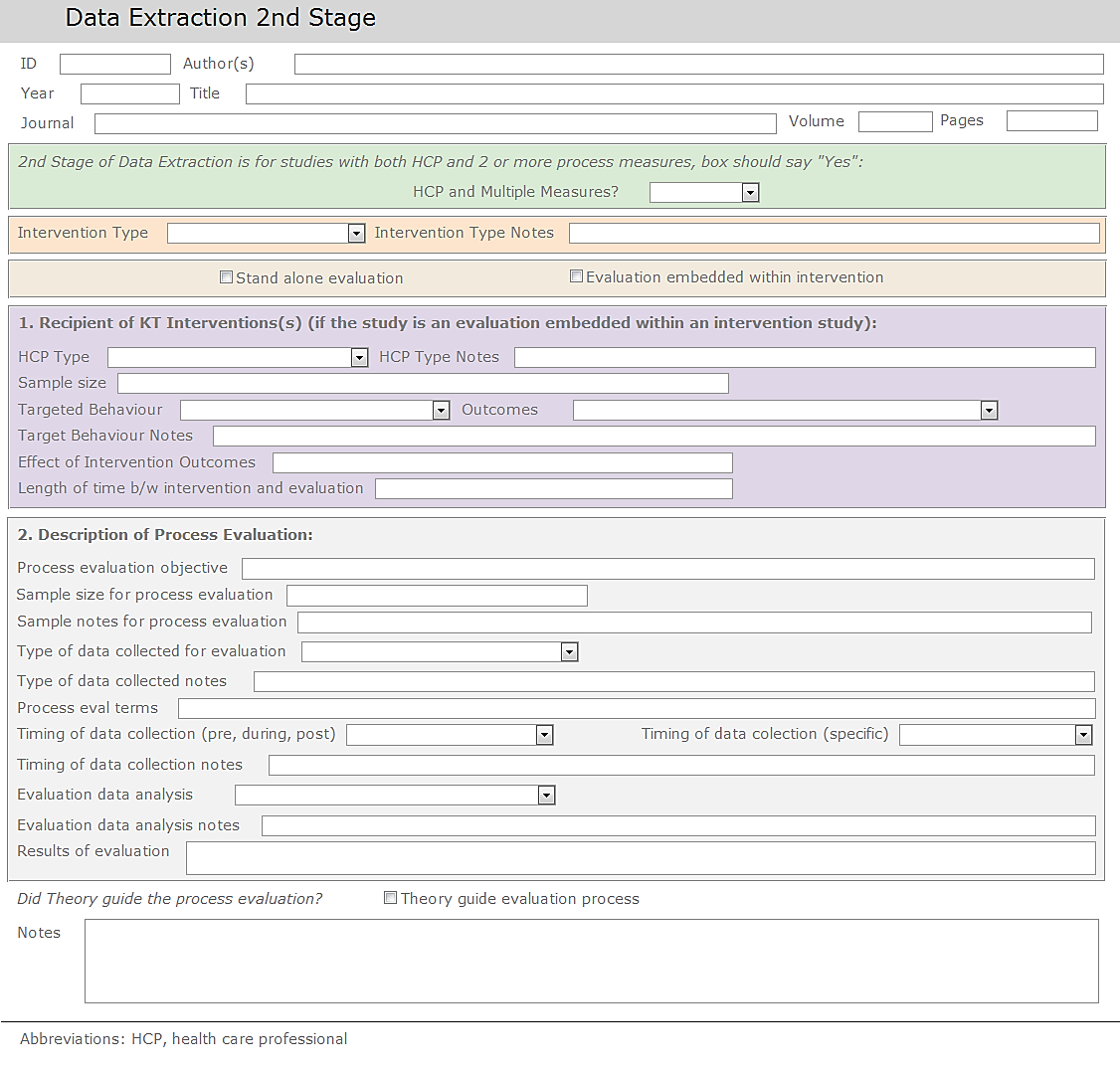

Supplement: Supplementary file 4 — Additional file 4: Standardized Microsoft Access data extraction form. Standardized Microsoft Access data extraction form to be used to extract data from included studies. (DOCX 327 KB) [file 13643_2014_316_MOESM4_ESM.docx]
