# Supplementary material for: A protocol for a systematic review of the use of process evaluations in knowledge translation research
Source: Syst Rev. 2014 Dec 23;3:149. doi: 10.1186/2046-4053-3-149 (PMC4307977; doi:10.1186/2046-4053-3-149)
Supplement: Supplementary file 3 — Additional file 3: Mixed methods appraisal tool (MMAT). Mixed methods quality assessment tool to be used to assess methodological quality of included research studies. (PDF 93 KB) [file 13643_2014_316_MOESM3_ESM.pdf]

McGill

Mixed Methods Appraisal Tool (MMAT) – Version 2011

For dissemination, application, and feedback: Please contact pierre.pluye@mcgill.ca, Department of Family Medicine, McGill University, Canada.

The MMAT is comprised of two parts (see below): criteria (Part I) and tutorial (Part II). While the content validity and the reliability of the pilot version of the MMAT have been examined, this critical appraisal tool is still in development. Thus, the MMAT must be used with caution, and users' feedback is appreciated. Cite the present version as follows.

> Pluye, P., Robert, E., Cargo, M., Bartlett, G., O'Cathain, A., Griffiths, F., Boardman, F., Gagnon, M.P., & Rousseau, M.C. (2011). *Proposal: A mixed methods appraisal tool for systematic mixed studies reviews.* Retrieved on [date] from http://mixedmethodsappraisaltoolpublic.pbworks.com. Archived by WebCite® at http://www.webcitation.org/5tTRTc9yJ

Purpose: The MMAT has been designed for the appraisal stage of complex systematic literature reviews that include qualitative, quantitative and mixed methods studies (mixed studies reviews). The MMAT permits to concomitantly appraise and describe the methodological quality for three methodological domains: mixed, qualitative and quantitative (subdivided into three sub-domains: randomized controlled, non-randomized, and descriptive). Therefore, using the MMAT requires experience or training in these domains. E.g., MMAT users may be helped by a colleague with specific expertise when needed. The MMAT allows the appraisal of most common types of study methodology and design. For appraising a qualitative study, use section 1 of the MMAT. For a quantitative study, use section 2 or 3 or 4, for randomized controlled, non-randomized, and descriptive studies, respectively. For a mixed methods study, use section 1 for appraising the qualitative component, the appropriate section for the quantitative component (2 or 3 or 4), and section 5 for the mixed methods component. For each relevant study selected for a systematic mixed studies review, the methodological quality can then be described using the corresponding criteria. This may lead to exclude studies with lowest quality from the synthesis, or to consider the quality of studies for contrasting their results (e.g., low quality vs. high).

Scoring metrics: For each retained study, an overall quality score may be not informative (in comparison to a descriptive summary using MMAT criteria), but might be calculated using the MMAT. Since there are only a few criteria for each domain, the score can be presented using descriptors such as *, **, ***, and ****. For qualitative and quantitative studies, this score can be the number of criteria met divided by four (scores varying from 25% (*) -one criterion met- to 100% (****) -all criteria met-). For mixed methods research studies, the premise is that the overall quality of a combination cannot exceed the quality of its weakest component. Thus, the overall quality score is the lowest score of the study components. The score is 25% (*) when *QUAL=1 or QUAN=1 or MM=0*; it is 50% (**) when *QUAL=2 or QUAN=2 or MM=1*; it is 75% (***) when *QUAL=3 or QUAN=3 or MM=2*; and it is 100% (****) when *QUAL=4 and QUAN=4 and MM=3* (QUAL being the score of the qualitative component; QUAN the score of the quantitative component; and MM the score of the mixed methods component).

Rationale: There are general criteria for planning, designing and reporting mixed methods research (Creswell and Plano Clark, 2010), but there is no consensus on key specific criteria for appraising the methodological quality of mixed methods studies (O'Cathain, Murphy and Nicholl, 2008). Based on a critical examination of 17 health-related systematic mixed studies reviews, an initial 15-criteria version of MMAT was proposed (Pluye, Gagnon, Griffiths and Johnson-Lafleur, 2009). This was pilot tested in 2009. Two raters assessed 29 studies using the pilot MMAT criteria and tutorial (Pace, Pluye, Bartlett, Macaulay et al., 2010). Based on this pilot exercise, it is anticipated that applying MMAT may take on average 15 minutes per study (hence efficient), and that the Intra-Class Correlation might be around 0.8 (hence reliable). The present 2011 revision is based on feedback from four workshops, and a comprehensive framework for assessing the quality of mixed methods research (O'Cathain, 2010).

Conclusion: The MMAT has been designed to appraise the *methodological quality* of the studies retained for a systematic mixed studies review, not the quality of their *reporting* (writing). This distinction is important, as good research may not be 'well' reported. If reviewers want to genuinely assess the former, companion papers and research reports should be collected when some criteria are not met, and authors of the corresponding publications should be contacted for additional information. Collecting additional data is usually necessary to appraise *qualitative research and mixed methods studies*, as there are no uniform standards for reporting study characteristics in these domains (www.equator-network.org), in contrast, e.g., to the CONSORT statement for reporting randomized controlled trials (www.consort-statement.org).

Authors and contributors: Pierre Pluye[1], Marie-Pierre Gagnon[2], Frances Griffiths[3] and Janique Johnson-Lafleur[1] proposed an initial version of MMAT criteria (Pluye et al., 2009). Romina Pace[1] and Pierre Pluye[1] led the pilot test. Gillian Bartlett[1], Belinda Nicolau[4], Robbyn Seller[1], Justin Jagosh[1], Jon Salsberg[1] and Ann Macaulay[1] contributed to the pilot work (Pace et al., 2010). Pierre Pluye[1], Émilie Robert[5], Margaret Cargo[6], Alicia O'Cathain[7], Frances Griffiths[3], Felicity Boardman[3], Marie-Pierre Gagnon[2], Gillian Bartlett[1], and Marie-Claude Rousseau[8] contributed to the present 2011 version.

Affiliations: 1. Department of Family Medicine, McGill University, Canada; 2. Faculté des sciences infirmières, Université Laval, Canada; 3. Warwick Medical School, University of Warwick, UK; 4. Faculty of Dentistry, McGill University, Canada; 5. Centre de recherche du CHUM, Université de Montréal, Canada; 6. School of Health Sciences, University of South Australia, Australia; 7. Medical Care Research Unit, ScHARR, University of Sheffield, UK; 8. INRS-Institut Armand Frappier, Laval, Canada.

PART I. MMAT criteria & one-page template (to be included in appraisal forms)

**Types of mixed methods study components or primary studies**	**Methodological quality criteria (see tutorial for definitions and examples)**	**Responses**			
		Yes	No	Can't tell	Comments
**Screening questions (for all types)**	• Are there clear qualitative and quantitative research questions (or objectives*), or a clear mixed methods question (or objective*)?				
	• Do the collected data allow address the research question (objective)? E.g., consider whether the follow-up period is long enough for the outcome to occur (for longitudinal studies or study components).				
	Further appraisal may be not feasible or appropriate when the answer is 'No' or 'Can't tell' to one or both screening questions.				
**1. Qualitative**	1.1. Are the sources of qualitative data (archives, documents, informants, observations) relevant to address the research question (objective)?				
	1.2. Is the process for analyzing qualitative data relevant to address the research question (objective)?				
	1.3. Is appropriate consideration given to how findings relate to the context, e.g., the setting, in which the data were collected?				
	1.4. Is appropriate consideration given to how findings relate to researchers' influence, e.g., through their interactions with participants?				
**2. Quantitative randomized controlled (trials)**	2.1. Is there a clear description of the randomization (or an appropriate sequence generation)?				
	2.2. Is there a clear description of the allocation concealment (or blinding when applicable)?				
	2.3. Are there complete outcome data (80% or above)?				
	2.4. Is there low withdrawal/drop-out (below 20%)?				
**3. Quantitative non-randomized**	3.1. Are participants (organizations) recruited in a way that minimizes selection bias?				
	3.2. Are measurements appropriate (clear origin, or validity known, or standard instrument; and absence of contamination between groups when appropriate) regarding the exposure/intervention and outcomes?				
	3.3. In the groups being compared (exposed vs. non-exposed; with intervention vs. without; cases vs. controls), are the participants comparable, or do researchers take into account (control for) the difference between these groups?				
	3.4. Are there complete outcome data (80% or above), and, when applicable, an acceptable response rate (60% or above), or an acceptable follow-up rate for cohort studies (depending on the duration of follow-up)?				
**4. Quantitative descriptive**	4.1. Is the sampling strategy relevant to address the quantitative research question (quantitative aspect of the mixed methods question)?				
	4.2. Is the sample representative of the population understudy?				
	4.3. Are measurements appropriate (clear origin, or validity known, or standard instrument)?				
	4.4. Is there an acceptable response rate (60% or above)?				
**5. Mixed methods**	5.1. Is the mixed methods research design relevant to address the qualitative and quantitative research questions (or objectives), or the qualitative and quantitative aspects of the mixed methods question (or objective)?				
	5.2. Is the integration of qualitative and quantitative data (or results*) relevant to address the research question (objective)?				
	5.3. Is appropriate consideration given to the limitations associated with this integration, e.g., the divergence of qualitative and quantitative data (or results*) in a triangulation design?				
	Criteria for the qualitative component (1.1 to 1.4), and appropriate criteria for the quantitative component (2.1 to 2.4, or 3.1 to 3.4, or 4.1 to 4.4), must be also applied.				

*These two items are not considered as double-barreled items since in mixed methods research, (1) there may be research questions (quantitative research) or research objectives (qualitative research), and (2) data may be integrated, and/or qualitative findings and quantitative results can be integrated.

PART II. MMAT tutorial

Types of mixed methods study components or primary studies	Methodological quality criteria
**1. Qualitative**<br><br>Common types of qualitative research methodology include:<br><br>A. Ethnography<br>The aim of the study is to describe and interpret the shared cultural behaviour of a group of individuals.<br><br>B. Phenomenology<br>The study focuses on the subjective experiences and interpretations of a phenomenon encountered by individuals.<br><br>C. Narrative<br>The study analyzes life experiences of an individual or a group.<br><br>D. Grounded theory<br>Generation of theory from data in the process of conducting research (data collection occurs first).<br><br>E. Case study<br>In-depth exploration and/or explanation of issues intrinsic to a particular case. A case can be anything from a decision-making process, to a person, an organization, or a country.<br><br>F. Qualitative description<br>There is no specific methodology, but a qualitative data collection and analysis, e.g., in-depth interviews or focus groups, and hybrid thematic analysis (inductive and deductive).<br><br>Key references: Creswell, 1998; Schwandt, 2001; Sandelowski, 2010.	**1.1. Are the sources of qualitative data (archives, documents, informants, observations) relevant to address the research question (objective)?**<br><br>E.g., consider whether (a) the selection of the participants is clear, and appropriate to collect relevant and rich data; and (b) reasons why certain potential participants chose not to participate are explained.
	**1.2. Is the process for analyzing qualitative data relevant to address the research question (objective)?**<br><br>E.g., consider whether (a) the method of data collection is clear (in depth interviews and/or group interviews, and/or observations and/or documentary sources); (b) the form of the data is clear (tape recording, video material, and/or field notes for instance); (c) changes are explained when methods are altered during the study; and (d) the qualitative data analysis addresses the question.
	**1.3. Is appropriate consideration given to how findings relate to the context, e.g., the setting, in which the data were collected?** *<br><br>E.g., consider whether the study context and how findings relate to the context or characteristics of the context are explained (how findings are influenced by or influence the context). "For example, a researcher wishing to observe care in an acute hospital around the clock may not be able to study more than one hospital. (…) Here, it is essential to take care to describe the context and particulars of the case [the hospital] and to flag up for the reader the similarities and differences between the case and other settings of the same type" (Mays & Pope, 1995).<br><br>The notion of context may be conceived in different ways depending on the approach (methodology) tradition.
	**1.4. Is appropriate consideration given to how findings relate to researchers' influence, e.g., through their interactions with participants?** *<br><br>E.g., consider whether (a) researchers critically explain how findings relate to their perspective, role, and interactions with participants (how the research process is influenced by or influences the researcher); (b) researcher's role is influential at all stages (formulation of a research question, data collection, data analysis and interpretation of findings); and (c) researchers explain their reaction to critical events that occurred during the study.<br><br>The notion of reflexivity may be conceived in different ways depending on the approach (methodology) tradition. E.g., "at a minimum, researchers employing a generic approach [qualitative description] must explicitly identify their disciplinary affiliation, what brought them to the question, and the assumptions they make about the topic of interest" (Caelli, Ray & Mill, 2003, p. 5).

*See suggestion on the MMAT wiki homepage (under '2011 version'): Independent reviewers can establish a common understanding of these two items prior to beginning the critical appraisal.

Types of mixed methods study components or primary studies	Methodological quality criteria
**2. Quantitative randomized controlled (trials)**<br><br>Randomized controlled clinical trial: A clinical study in which individual participants are allocated to intervention or control groups by randomization (intervention assigned by researchers).<br><br>Key references: Higgins & Green, 2008; Porta, 2008; Oxford Center for Evidence based medicine, 2009.	**2.1. Is there a clear description of the randomization (or an appropriate sequence generation)?**<br><br>In a randomized controlled trial, the allocation of a participant (or a data collection unit, e.g., a school) into the intervention or control group is based solely on chance, and researchers describe how the randomization schedule is generated. “A simple statement such as ‘we randomly allocated’ or ‘using a randomized design’ is insufficient”.<br><br>*Simple randomization:* Allocation of participants to groups by chance by following a predetermined plan/sequence. “Usually it is achieved by referring to a published list of random numbers, or to a list of random assignments generated by a computer”.<br><br>*Sequence generation:* “The rule for allocating interventions to participants must be specified, based on some chance (random) process”. Researchers provide sufficient detail to allow a readers’ appraisal of whether it produces comparable groups. E.g., blocked randomization (to ensure particular allocation ratios to the intervention groups), or stratified randomization (randomization performed separately within strata), or minimization (to make small groups closely similar with respect to several characteristics).
	**2.2. Is there a clear description of the allocation concealment (or blinding when applicable)?**<br><br>*The allocation concealment protects assignment sequence until allocation.* E.g., researchers and participants are unaware of the assignment sequence up to the point of allocation. E.g., group assignment is concealed in opaque envelops until allocation.<br><br>*The blinding protects assignment sequence after allocation.* E.g., researchers and/or participants are unaware of the group a participant is allocated to during the course of the study.
	**2.3. Are there complete outcome data (80% or above)?**<br><br>E.g., almost all the participants contributed to almost all measures.
	**2.4. Is there low withdrawal/drop-out (below 20%)?**<br><br>E.g., almost all the participants completed the study.

Types of mixed methods study components or primary studies	Methodological quality criteria
**3. Quantitative non-randomized**<br><br>Common types of design include (A) non-randomized controlled trials, and (B-C-D) observational analytic study or component where the intervention/exposure is defined/assessed, but not assigned by researchers.<br><br>A. Non-randomized controlled trials<br>The intervention is assigned by researchers, but there is no randomization, e.g., a pseudo-randomization. A non-random method of allocation is not reliable in producing alone similar groups.<br><br>B. Cohort study<br>Subsets of a defined population are assessed as exposed, not exposed, or exposed at different degrees to factors of interest. Participants are followed over time to determine if an outcome occurs (prospective longitudinal).<br><br>C. Case-control study<br>Cases, e.g., patients, associated with a certain outcome are selected, alongside a corresponding group of controls. Data is collected on whether cases and controls were exposed to the factor under study (retrospective).<br><br>D. Cross-sectional analytic study<br>At one particular time, the relationship between health-related characteristics (outcome) and other factors (intervention/exposure) is examined. E.g., the frequency of outcomes is compared in different population sub-groups according to the presence/absence (or level) of the intervention/exposure.<br><br>Key references for observational analytic studies: Higgins & Green, 2008; Wells, Shea, O'Connell, Peterson, et al., 2009.	**3.1. Are participants (organizations) recruited in a way that minimizes selection bias?**<br><br>At recruitment stage:<br><br>For cohort studies, e.g., consider whether the exposed (or with intervention) and non-exposed (or without intervention) groups are recruited from the same population.<br>For case-control studies, e.g., consider whether same inclusion and exclusion criteria were applied to cases and controls, and whether recruitment was done independently of the intervention or exposure status.<br>For cross-sectional analytic studies, e.g., consider whether the sample is representative of the population.
	**3.2. Are measurements appropriate (clear origin, or validity known, or standard instrument; and absence of contamination between groups when appropriate) regarding the exposure/intervention and outcomes?**<br><br>At data collection stage:<br><br>E.g., consider whether (a) the variables are clearly defined and accurately measured; (b) the measurements are justified and appropriate for answering the research question; and (c) the measurements reflect what they are supposed to measure.<br><br>For non-randomized controlled trials, the intervention is assigned by researchers, and so consider whether there was absence/presence of a contamination. E.g., the control group may be indirectly exposed to the intervention through family or community relationships.
	**3.3. In the groups being compared (exposed vs. non-exposed; with intervention vs. without; cases vs. controls), are the participants comparable, or do researchers take into account (control for) the difference between these groups?**<br><br>At data analysis stage:<br><br>For cohort, case-control and cross-sectional, e.g., consider whether (a) the most important factors are taken into account in the analysis; (b) a table lists key demographic information comparing both groups, and there are no obvious dissimilarities between groups that may account for any differences in outcomes, or dissimilarities are taken into account in the analysis.
	**3.4. Are there complete outcome data (80% or above), and, when applicable, an acceptable response rate (60% or above), or an acceptable follow-up rate for cohort studies (depending on the duration of follow-up)?**

Types of mixed methods study components or primary studies	Methodological quality criteria
**4. Quantitative descriptive studies**<br><br>Common types of design include single-group studies:<br><br>A. Incidence or prevalence study without comparison group<br>In a defined population at one particular time, what is happening in a population, e.g., frequencies of factors (importance of problems), is described (portrayed).<br><br>B. Case series<br>A collection of individuals with similar characteristics are used to describe an outcome.<br><br>C. Case report<br>An individual or a group with a unique/unusual outcome is described in details.<br><br>Key references: Critical Appraisal Skills Programme, 2009; Draugalis, Coons & Plaza, 2008.	**4.1. Is the sampling strategy relevant to address the quantitative research question (quantitative aspect of the mixed methods question)?**<br><br>E.g., consider whether (a) the source of sample is relevant to the population under study; (b) when appropriate, there is a standard procedure for sampling, and the sample size is justified (using power calculation for instance).
	**4.2. Is the sample representative of the population understudy?**<br><br>E.g., consider whether (a) inclusion and exclusion criteria are explained; and (b) reasons why certain eligible individuals chose not to participate are explained.
	**4.3. Are measurements appropriate (clear origin, or validity known, or standard instrument)?**<br><br>E.g., consider whether (a) the variables are clearly defined and accurately measured; (b) measurements are justified and appropriate for answering the research question; and (c) the measurements reflect what they are supposed to measure.
	**4.4. Is there an acceptable response rate (60% or above)?**<br><br>The response rate is not pertinent for case series and case report. E.g., there is no expectation that a case series would include all patients in a similar situation.

Types of mixed methods study components or primary studies	Methodological quality criteria
**5. Mixed methods**<br><br>Common types of design include:<br><br>A. Sequential explanatory design<br>The quantitative component is followed by the qualitative. The purpose is to explain quantitative results using qualitative findings. E.g., the quantitative results guide the selection of qualitative data sources and data collection, and the qualitative findings contribute to the interpretation of quantitative results.<br><br>B. Sequential exploratory design<br>The qualitative component is followed by the quantitative. The purpose is to explore, develop and test an instrument (or taxonomy), or a conceptual framework (or theoretical model). E.g., the qualitative findings inform the quantitative data collection, and the quantitative results allow a generalization of the qualitative findings.<br><br>C. Triangulation design<br>The qualitative and quantitative components are concomitant. The purpose is to examine the same phenomenon by interpreting qualitative and quantitative results (bringing data analysis together at the interpretation stage), or by integrating qualitative and quantitative datasets (e.g., data on same cases), or by transforming data (e.g., quantization of qualitative data).<br><br>D. Embedded design<br>The qualitative and quantitative components are concomitant. The purpose is to support a qualitative study with a quantitative sub-study (measures), or to better understand a specific issue of a quantitative study using a qualitative sub-study, e.g., the efficacy or the implementation of an intervention based on the views of participants.<br><br>Key references: Creswell & Plano Clark, 2007; O'Cathain, 2010.	**5.1. Is the mixed methods research design relevant to address the qualitative and quantitative research questions (or objectives), or the qualitative and quantitative aspects of the mixed methods question (or objective)?**<br><br>E.g., the rationale for integrating qualitative and quantitative methods to answer the research question is explained.<br><br>**5.2. Is the integration of qualitative and quantitative data (or results) relevant to address the research question (objective)?**<br><br>E.g., there is evidence that data gathered by both research methods was brought together to form a complete picture, and answer the research question; authors explain when integration occurred (during the data collection-analysis or/and during the interpretation of qualitative and quantitative results); they explain how integration occurred and who participated in this integration.<br><br>**5.3. Is appropriate consideration given to the limitations associated with this integration, e.g., the divergence of qualitative and quantitative data (or results)?**

References

- Caelli, K., Ray, L., & Mill, J. (2003). 'Clear as Mud': Toward greater clarity in generic qualitative research. *International Journal of Qualitative Methods, 2(*2), 1-23.
- Creswell, J., & Plano Clark, V. (2007). *Designing and conducting mixed methods research*. London: Sage.
- Creswell, J. (1998). *Qualitative Inquiry and Research Design: Choosing Among Five Approaches*. Thousand Oaks: Sage.
- Critical Appraisal Skills Programme (2009). CASP appraisal tools. Retrieved on August 26, 2009 from: *www.phru.nhs.uk/pages/PHD/resources.htm*
- Draugalis, J.R., Coons, S.J., & Plaza, C.M. (2008). Best practices for survey research reports: a synopsis for authors and reviewers. *American Journal of Pharmaceutical Education, 72(1),* e11.
- Higgins, J.P.T. & Green, S. (2008). *Cochrane Handbook for Systematic Reviews of Interventions* - Version 5.0.1 [updated September 2008]. The Cochrane Collaboration. Retrieved on August 26, 2009 from www.cochrane-handbook.org
- Mays, N., & Pope, C. (1995). Qualitative Research: Rigour and qualitative research. *British Medical Journal, 311(6997),* 109-112.
- O'Cathain, A., Murphy, E. & Nicholl, J. (2008). The quality of mixed methods studies in health services research. *Journal of Health Services Research and Policy, 13(2),* 92-98.
- O'Cathain, A. (2010). Assessing the quality of mixed methods research: Towards a comprehensive framework. In A. Tashakkori & C. Teddlie (Eds.), *Handbook of mixed methods in social and behavioral research (2nd edition)* (pp. 531-555). Thousand Oaks: Sage.
- Pace, R., Pluye, P., Bartlett, G., Macaulay, A., Salsberg, J., Jagosh, J., & Seller, R. (2010). *Reliability of a tool for concomitantly appraising the methodological quality of qualitative, quantitative and mixed methods research: a pilot study*. 38th Annual Meeting of the North American Primary Care Research Group (NAPCRG), Seattle, USA.
- Pluye, P., Gagnon, M.P., Griffiths, F. & Johnson-Lafleur, J. (2009). A scoring system for appraising mixed methods research, and concomitantly appraising qualitative, quantitative and mixed methods primary studies in Mixed Studies Reviews. *International Journal of Nursing Studies, 46(4),* 529-46.
- Oxford Center for Evidence Based Medicine (2009). Levels of evidence. Retrieved on July 7, 2009 from www.cebm.net/levels_of_evidence.asp
- Porta, M. (2008). *A Dictionary of Epidemiology*. New York: Oxford University Press.
- Sandelowski, M. (2010). What's in a name? Qualitative description revisited. *Research in Nursing and Health, 33*(1), 77-84.
- Schwandt, T. (2001). *Dictionary of qualitive inquiry*. Thousand Oaks: Sage.
- Wells, G.A., Shea, B., O'Connell, D., Peterson, J., Welch, V., Losos, M., & Tugwell, P. (2009). *The Newcastle-Ottawa Scale (NOS) for assessing the quality of non-randomised studies in meta-analyses*. The Cochrane Non-Randomized Studies Method Group. Retrieved on July 7, 2009 from www.ohri.ca/programs/clinical_epidemiology/oxford.htm
